# Supplementary figures and images for: Characterisation of the RNA Virome of Nine Ochlerotatus Species in Finland
Source: Viruses. 2022 Jul 7;14(7):1489. doi: 10.3390/v14071489 (PMC9324324; doi:10.3390/v14071489)

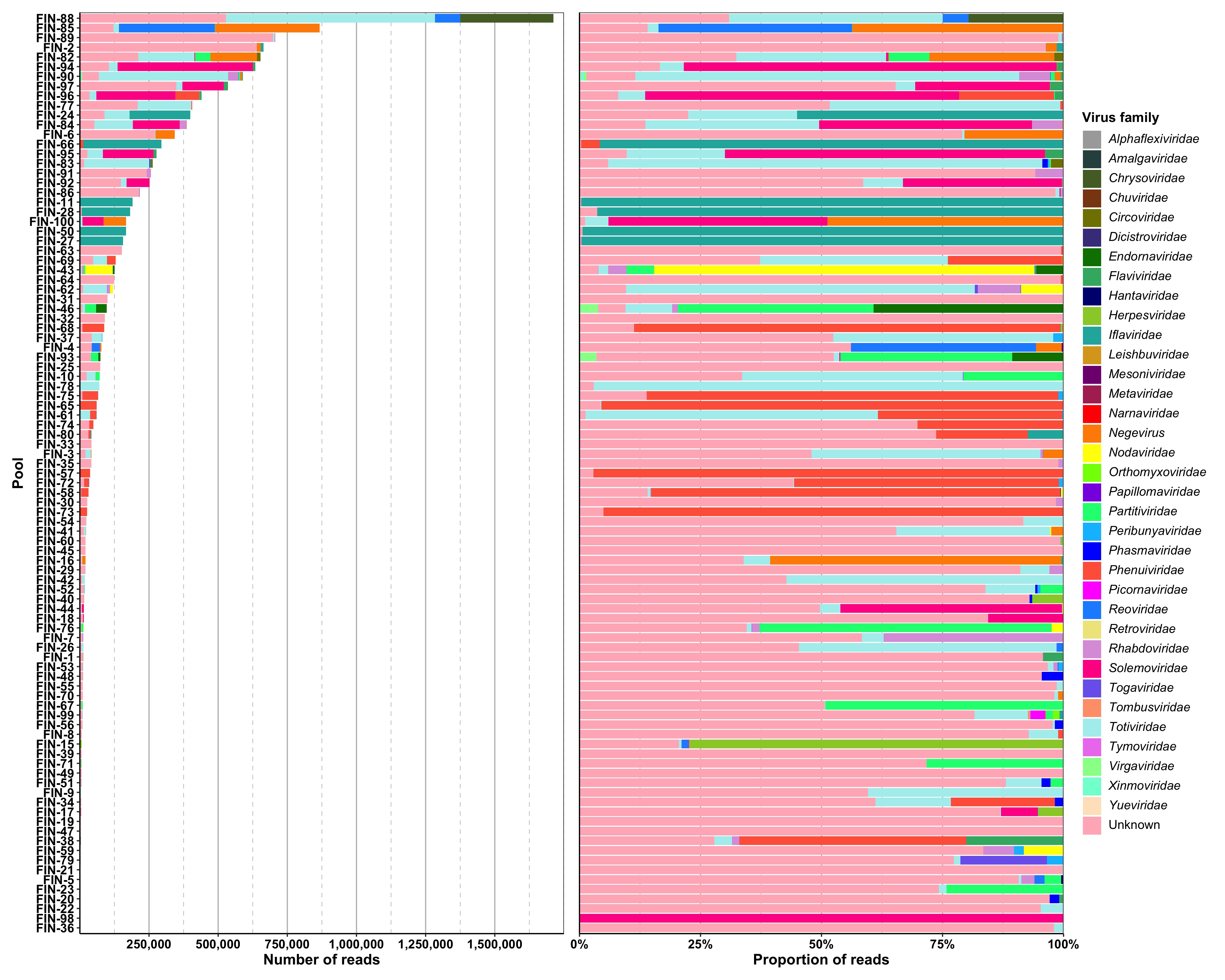

Supplement: Supplementary file 1 [file viruses-14-01489-s001.zip › Figure_S1.jpeg]

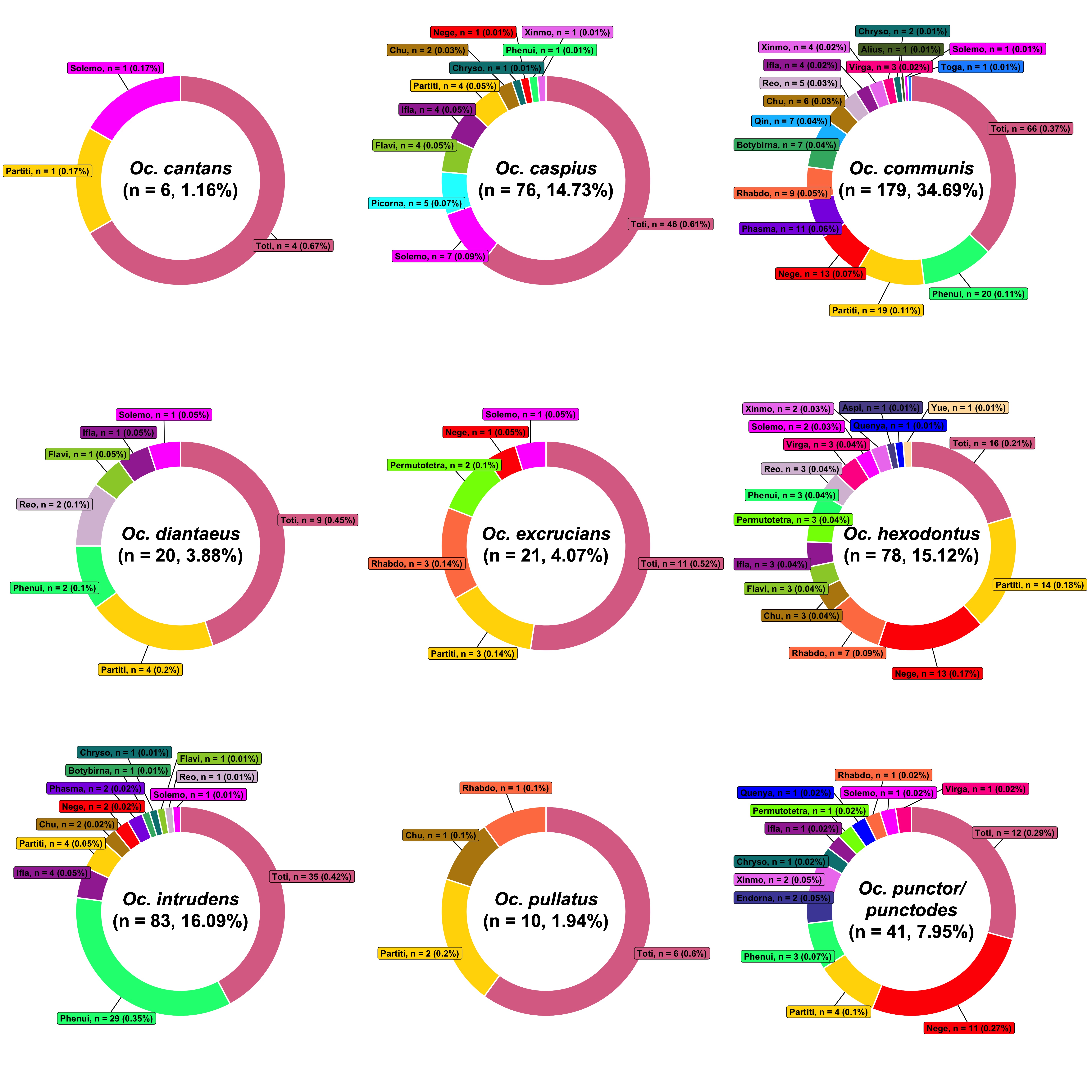

Supplement: Supplementary file 1 [file viruses-14-01489-s001.zip › Figure_S3.jpeg]
